# Surgeon Workforce in Underserved Communities

**DOI:** 10.1001/jamahealthforum.2024.3531

**Published:** 2024-11-01

**Authors:** Crystal D. Taylor, Sara L. Schaefer, Adrian Diaz, Nicholas Kunnath, John W. Scott, Andrew M. Ibrahim

**Affiliations:** 1 Center for Healthcare Outcomes & Policy, University of Michigan, Ann Arbor; 2 Department of Surgery, University of Chicago, Chicago, Illinois; 3 Department of Surgery, University of Washington, Seattle; 4 Institute for Health Metrics and Evaluation, University of Washington, Seattle

## Abstract

This cohort study evaluates the Health Professional Shortage Area Surgeon Incentive Program to determine how it may have changed the surgeon workforce in designated areas.

## Introduction

The Health Professional Shortage Area (HPSA) designation was created by the Health Resources and Services Administration to identify communities that lack clinical health care professionals. Areas with the designation are eligible for financial incentives from the Centers for Medicare & Medicaid Services as a way to attract more health care professionals. While the program historically focused on primary care and mental health professionals,^1^ in 2011 surgeons were added for a trial period up to 2015. Through the HPSA Surgeon Incentive Program (SIP), general surgeons providing care at facilities with HPSA designation would be eligible for a 10% payment bonus.^2^ Despite the policy being established more than a decade ago, there is still a limited understanding of how it may have changed the surgeon workforce in these shortage areas. Evaluation of the SIP takes on timely importance as calls have been made for reauthorization of the policy and related workforce legislation pending in the US Congress.^3,4^

## Methods

Data from the Health Resources and Services Administration were used to identify the number of general surgeons per 100 000 residents in 583 SIP-eligible counties. We performed a difference-in-differences analysis to evaluate changes in surgeon workforce within SIP-eligible counties vs non–SIP-eligible counties. Data included annual surgeon workforce by county from 2010 to 2020, which includes time periods before, during, and after the SIP, which was effective from 2011 to 2015. To better understand the potential heterogeneity of treatment effect of the policy during this same time frame, we also geospatially evaluated each individual county comparing changes in workforce over the study period. This study was deemed exempt by the University of Michigan institutional review board. We followed the STROBE reporting guidelines for cohort studies. Data were analyzed from September to October 2023.

## Results

**Figure 1.**
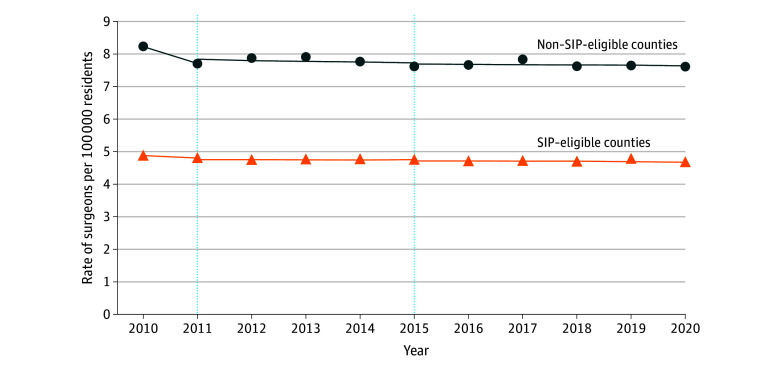
Difference-in-Differences Analysis of the Surgeon Incentive Program (SIP) in SIP-Eligible and Non–SIP-Eligible Counties The dotted lines represent when the SIP began in 2011 and ended in 2015. At baseline in the prepolicy period, non–SIP-eligible counties had higher rates of general surgeons than SIP-eligible counties. The general surgeon density remained relatively constant across the study period.

**Figure 2.**
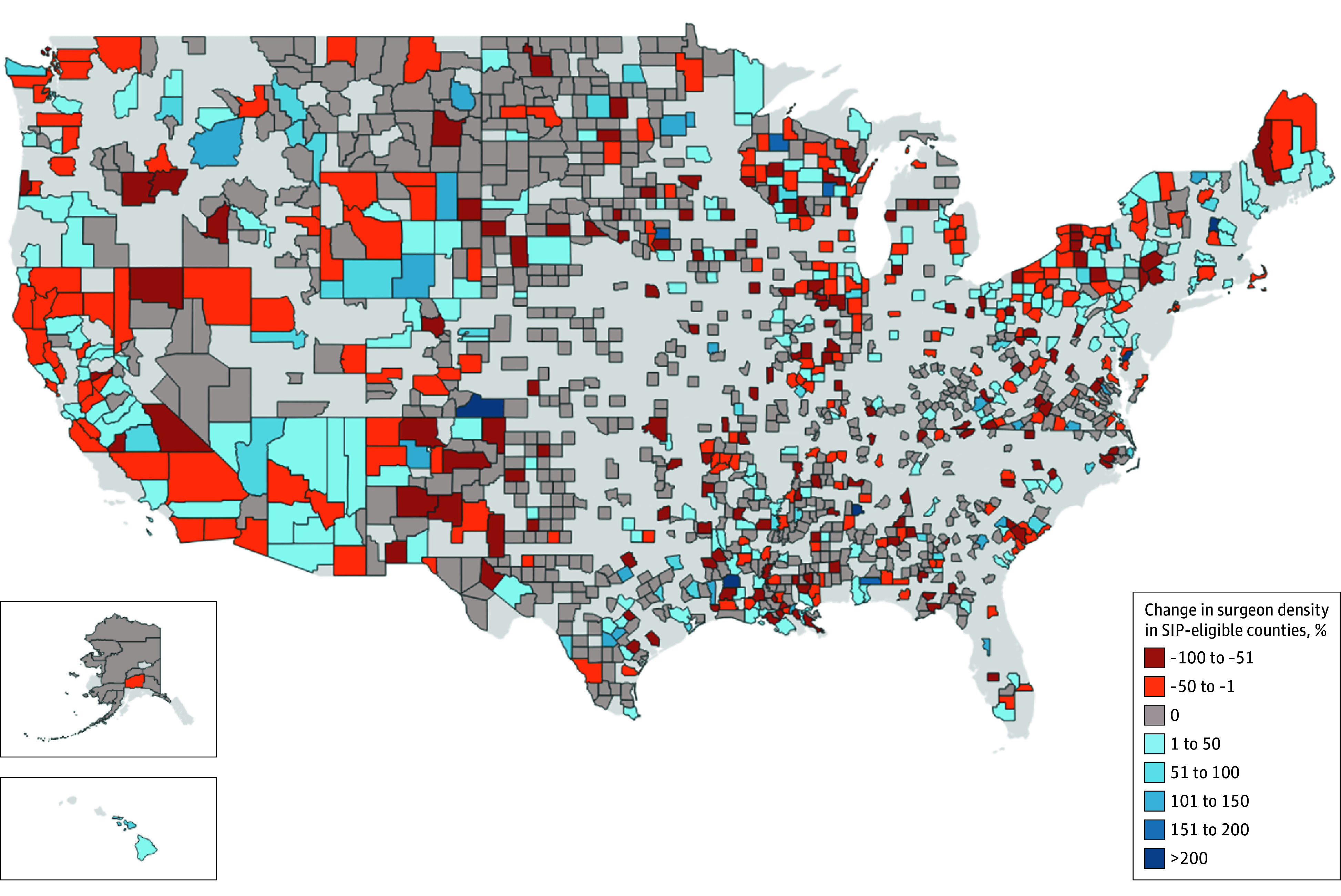
Change in Surgeon Density in Eligible Counties During and After the Surgeon Incentive Program (SIP) Orange and red areas demonstrate a decrease in surgeon density in select counties, while blue areas demonstrate an increase in surgeon density. Gray areas represent counties that experienced no change in surgeon density. The observed changes are heterogenous.

At the start of the study period in 2010, SIP-eligible counties on average had 8.2 surgeons per 100 000 residents compared to non–SIP-eligible counties that had 4.9 surgeons per 100 000 residents. From 2011 to 2015, the difference in differences between the 2 groups was 0.05 surgeons per 100 000 residents, with no statistically significant change afterward (Figure 1). Of the 583 SIP-eligible counties evaluated, 81 counties (13.9%) increased the number of general surgeons practicing in these shortage areas, 80 counties (13.7%) decreased, and 422 counties (72.4%) experienced no change (Figure 2).

## Discussion

This evaluation of the SIP has 2 principal findings. First, and overall, there appears to be no statistically significant differences in surgeon workforce during or after the payment incentive. Second, a more granular geospatial evaluation suggests heterogeneity of policy effect, with a portion of eligible counties demonstrating statistically significant increases in surgeon workforce while others saw notable decreases. Taken together, a better understanding of why the policy had variable effects on the surgeon workforce would be informative to future proposals to reinstate the SIP or enact a similar policy. The Ensuring Access to General Surgery Act is being actively considered with bipartisan support in both the Senate and House to study whether HPSA designation accurately assesses access to general surgeons.^4^ In addition, we propose including engagement with surgeons and primary care physicians,^5^ who both collectively shape access to surgical care. In doing so, synergistic overlaps of primary care and surgeon HPSAs can be identified.

A limitation of this study relates to the lack of granular data. Specifically, we were not able to determine why certain counties saw increases vs decreases in their workforce during this study period. However, the data we used are the same as those used by congressional leaders to make workforce policy and mirrors prior studies evaluating health care workforce distribution.^6^

## Notes

Supplement. Data Sharing Statement
