# Supplementary material for: Surgeon Workforce in Underserved Communities
Source: JAMA Health Forum. Author manuscript; Available in PMC 2024 Nov 23. (PMC11581652; doi:10.1001/jamahealthforum.2024.3531)
Supplement: Data Sharing Agreement [file NIHMS2033355-supplement-Data_Sharing_Agreement.pdf]

Data Sharing Statement

Taylor. Surgeon Workforce in Underserved Communities. *JAMA Health Forum*. Published November 01, 2024. doi:10.1001/jamahealthforum.2024.3531

Data
Data available: No

Additional Information
Explanation for why data not available: We used publicly available data from the Health Resources and Services Administration.
